# Facile Synthesis of Novel Prussian Blue–Lipid Nanocomplexes

**DOI:** 10.3390/molecules24224137

**Published:** 2019-11-15

**Authors:** Maria Antònia Busquets, Ariadna Novella-Xicoy, Valeria Guzmán, Joan Estelrich

**Affiliations:** 1 Pharmacy and Pharmaceutical Technology and, Physical Chemistry Department, Faculty of Pharmacy and Food Sciences, University of Barcelona, Avda. Joan XXIII, 27-31, 08028 Barcelona, Catalonia, Spain; mabusquetsvinas@ub.edu (M.A.B.); anovelxi7@alumnes.ub.edu (A.N.-X.); 2 Institute of Nanoscience and Nanotechnology, IN^2^UB, Diagonal 645, 08028 Barcelona, Catalonia, Spain; 3 Department of Biotechnology, Polytechnic University of Sinaloa, Carretera Municipal Libre Mazatlán Higueras Km 3, 82199 Mazatlán, Sinaloa, Mexico; 2015030475@upsin.edu.mx

**Keywords:** Prussian blue nanoparticles, lipid nanoparticles, photothermal agent, photothermal therapy, NIR

## Abstract

Prussian blue (PB) is known for its multiple applications ranging from fine arts to therapeutics. More recently, PB nanoparticles have been pointed to as appealing photothermal agents (PA) when irradiated with wavelengths corresponding to the biological windows, namely regions located in the near infrared (NIR) zone. In addition, the combination of PB with other components such as phospholipids boosts their therapeutical potential by facilitating, for instance, the incorporation of drugs becoming suitable drug delivery systems. The novelty of the research relies on the synthesis procedure and characterization of hybrid lipid–PB nanoparticles with a high yield in a friendly environment suitable for photothermal therapy. This goal was achieved by first obtaining insoluble PB coated with oleylamine (OA) to facilitate its combination with lipids. The resulting lipid–PB complex showed a monomodal distribution of sizes with an overall size of around 100 nm and a polydispersity index of about 0.200. It highlights one critical step in the synthesis procedure that is the shaking time of the mixture of PB–OA nanoparticles with the lipid, which was found to be 48 h. This time assured homogeneous preparation without the need of further separation stages. Samples were stable for more than three months under several storage conditions.

## 1. Introduction

Prussian blue (PB) is a mixed-valence transition metal hexacyanoferrate that possesses a distinct blue color. Chemically, it is Fe_4_[Fe(CN)_6_]_3_ (IUPAC reference: Iron (II,III) hexacyanoferrate (II,III)) and is prepared from an Fe^3+^ salt and Fe^2+^(CN)_6_]^4−^ (the same final compound, but that obtained from an Fe^2+^ salt and Fe^3+^(CN)_6_]^3−^ is known as Turnbull’s blue) [1]. Depending on the exact stoichiometry and conditions of preparation, the chemical formula of PB can range from Fe^III^_4_[Fe^II^(CN)_6_]_3_·x H_2_O (x = 14–16) and KFe ^III^ [Fe^II^(CN)_6_]·xH_2_O (x = 1–5) [2], which are commonly called “insoluble” and “soluble PB”, respectively. PB can act as an ion exchange material, as it has high affinity for certain “soft” metal cations. This feature makes PB an antidote of choice in the case of poisoning due to certain kinds of heavy metals. For instance, PB, orally administered, adsorbs thallium and radioactive isotopes of cesium (in particular, the isotope ^137^Cs^+^) [3]. A guarantee on the suitability of the human use of PB is their approval by the Food and Drug Administration (FDA) as a material with good safety. Moreover, it is found in the List of Essential Medicines of the World Health Organization [4]. Very recently, nanoparticles composed of PB have been shown to possess important applications. They can act as catalyzers mimicking peroxidases [5] or for enhancing the detection of hydrogen peroxide by electrochemical methods [6]. PB nanoparticles associated with gadolinium constitute molecular imaging agents that can be detected by both fluorescence and magnetic resonance imaging (MRI) [7]. However, one of the most promising applications of PB nanoparticles is their use in photothermal therapy (PTT) as a new family of photothermal agents (PA) [8]. PTT is a suitable technique for treating diseases, especially cancer and bacterial infections. In both cases, the basis is the rise in temperature triggered by light [9]. This thermal effect, known as thermal ablation, characterized by temperatures above 50 °C for a short period of time (4–6 min) [10], causes denaturation of proteins and disruption of cell membranes in the tumor areas, resulting in severe damage to cancer cells. PTT is, indeed, a non-invasive and effective modality for solid tumor treatment. To achieve the high temperatures, the energy of an electromagnetic radiation belonging to the near infrared (NIR) zone is converted into heat. The PA, previously introduced into the tumor, performs this transduction. The successful efficacy and selectivity of photothermal ablation requires the presence of PA with flawless optical absorbance in the NIR region [11]. The number of nanoparticles, both organic and inorganic, suitable as PA is increasing significantly [12,13]. Among the inorganic substances, gold non-spherical nanoparticles (such as nanorods, nanostars, or nanoshells) are considered the most common and efficient nanoparticles for PTT [14,15]. In addition to the large absorption for wavelengths within the NIR region, the PA to be used must fulfill some requirements to achieve nanoparticle-based selective and efficient photothermal therapies; that is, easy functionalization, low toxicity, and biocompatibility. PB presents strong absorbance in the NIR region [16,17,18] and, moreover, easily attains the above requirements, especially in the case of water-soluble PB nanoparticles (e.g., PB nanoparticles coated with citric acid as a capping agent [19]). Recent findings in clinical therapy have proven an enhanced anti-cancer efficacy by combining classical chemotherapy with thermotherapy owing to the stimulated release of the drug into the tumor as a result of hyperthermia [20]. To this end, new drug delivery systems have been optimized with external triggering mechanisms, where the stimulus can be both endogenous (i.e., enzymatic or pH changes) or exogenous (i.e., light irradiation, magnetic field, temperature) [21]. Liposomes provide excellent biocompatibility and the ability to be engineered to respond to various types of release mechanisms [22]. In this way, light-to-heat conversion with a PA can be an appropriate tool to trigger drug release from liposomes. Generally, such liposomes bear doxorubicin in the aqueous inner pool as a typical anti-tumor drug model, and a PA in the bilayer [23,24,25]. As is well documented in literature [26], the insoluble PB solid synthesized from an aqueous mixture of Fe^III/II^ and [Fe^II/III^(CN)_6_]^4−/3−^, based on the total number of Fe, unavoidably contains 15% of active surface reaction sites, Fe^III^-OH_2_, on the basis. Taking into consideration this fact, another strategy has been developed. It consists of transforming the insoluble PB into dispersed solutions of PB nanoparticles (PB-NPs). First, the PB surface is modified with oleylamine via Fe^III^-OH_2_ sites. Then, the resulting hydrophobic nanoparticles are transformed to water-dispersible nanoparticles by modification of the surface with 1,2-distearoyl-sn-glycero-3-phosphoethanolamine-*N*-[amino (polyethylene glycol)-2000] (DSPE-PEG 2000) lipid through a thin film hydration process [26,27]. In this approach, doxorubicin, after being transformed into a hydrophobic molecule, is incorporated into the lipid portions of the resulting nanocomplex.

The objective of our study was the preparation and characterization of a new theranostic nanomedicine by mixing phospholipids with PB. Phospholipids, with their amphiphilicity and diversity of head and tail chemistry, present anti-fouling properties that block unspecific binding to lipid-coated surfaces. They are a powerful toolbox for nanotechnology [28]. The mixing of phospholipids with PB, although it does not contribute to the therapeutic or diagnostic payload, increases the bioavailability of the nanocomplex. PB has already been encapsulated in liposomes as a nanosystem to absorb toxic cations [29]. In this work, liposomes incorporated an antibiotic ion channel as a vessel for synthesizing PB within the liposomes. Here, we describe a relatively simple method to obtain lipid structures bearing PB nanoparticles. The first challenge to achieve the incorporation of PB in the lipid structures is the transformation of insoluble PB in a product dispersible in lipids. This is reached by the modification of the surface of PB nanoparticles with oleylamine. The solubilization in organic media is afforded by the layer of oleylamine that coats the nanoparticles. Using this approach, we modified the surface of PB nanoparticles with oleylamine, yielding stable PB nanoparticles (OA–PB) easily dispersible into usual organic solvents. Thus, OA–PB nanoparticles can be mixed with the lipid solution to further form a PB-containing lipid complex (L–OA–PB). The obtained hybrid nanomaterial presents similarities with liposomes (same lipid composition), but also important differences (in our nanocomplex, phospholipids are not assembled into bilayers). It could be used for a combined treatment of cancer; that is, the nanocomplex obtained will able to bear a cytostatic agent (for instance, doxorubicin) and, in this way, the photothermal activity of PB nanoparticles will be completed with the chemotherapy achieved by the drug. Thus, we have created a lípid nanocomplex using only safe, biodegradable, and biocompatible materials. The nanocomplex presents a clear therapeutic approach, photothermal therapy, and, occasionally, this approach could be combined with chemotherapy.

## 2. Results and Discussion

### 2.1. Characteristics of PB Nanoparticles

**Figure 1:**
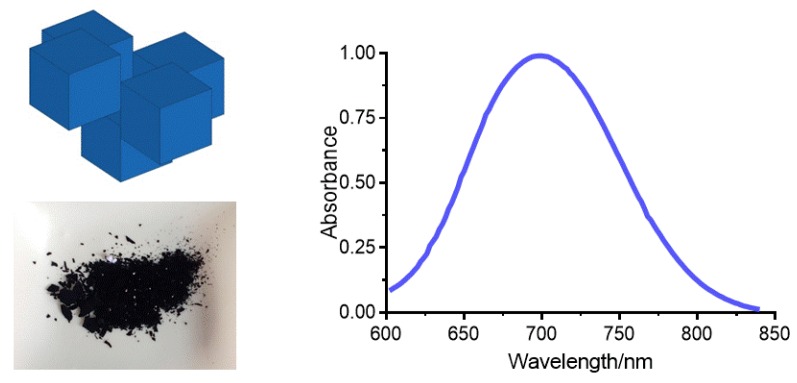
(**Left**) Supposed cubic structure of Prussian blue (PB) nanoparticles, and powder of PB obtained after grinding with an agate mortar. (**Right**) Visible spectrum of PB nanoparticles.

After precipitation, a dark blue powder was obtained (Figure 1, left). Although the insoluble PB appears to be a bulk blue powder, it could actually be regarded as an aggregate formed by nanoparticles of few nanometers [30]. The blue color of PB nanoparticles results from a charge transfer (CT) transition, that is, to the transfer of one electron from the Fe^II^ centers to the Fe^III^ centers [31]. The absorption spectrum of PB afforded a band centered at 700 nm (Figure 1, right). The wavelength of the maximum was independent of the PB nanoparticles concentration. In the Tris buffer (10 mM, pH 7.4), a molar absorptivity of 6367 ± 112 M^−1^ cm^−1^ was obtained.

**Figure 2:**
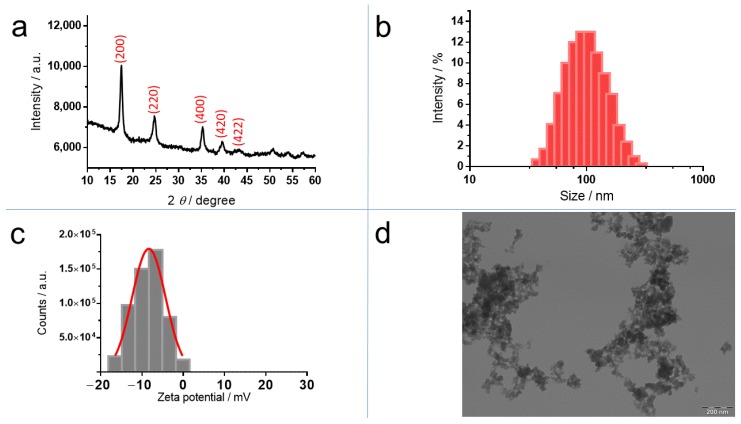
Characterization of PB nanoparticles: (**a**) X–Ray diffraction (XRD) pattern; (**b**) size distribution determined by dynamic light scattering (**c**) ζ-potential distribution measured by Doppler electrophoresis and (**d**) transmission electron microscopy (TEM) image.

XRD analysis of the powder (Figure 2a) showed broad peaks at 17.6° (200), 24.8° (220), 35.2° (400), 39.6° (420), and 43.5° (422), which can be indexed to the PB cubic structure Fe_4_[Fe(CN)_6_]_3_ (JCPDS 73-0687) and the space group Fm3m [32]. The mean particle size of the crystal was calculated from Scherrer’s equation using the width of the peak at 2θ = 17.6°. This size was estimated as ~10 nm.

The hydrodynamic diameter of PB in water was 109.7 ± 7.8 nm, which is an average value from five measurements determined by dynamic light scattering (DLS). The polydispersity index was 0.367 ± 0.046 (Figure 2b). The ζ-potential value of PB in KCl 10^−3^ M was –9.7 ± 0.4 mV (*n* = 5) (Figure 2c). The TEM images of PB obtained according the described method, that is, at room temperature (RT), revealed that PB was an aggregated form of nanoparticles of 10–20 nm (Figure 2d). This value was concordant with the average PB nanoparticles’ size about 10 nm by estimating the crystallite size from Scherrer’s equation.

**Figure 3:**
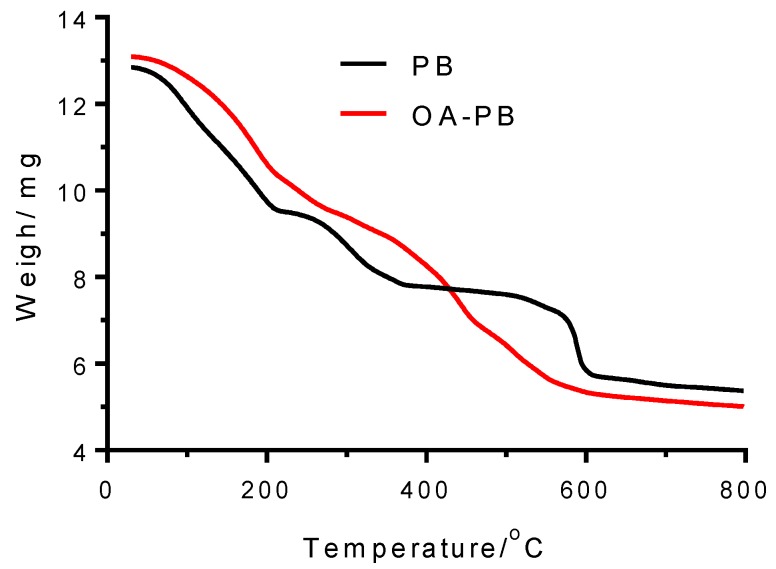
Thermogravimetric analysis of PB samples carried out in nitrogen atmosphere. Black line: Bulk PB (PB); red line: PB coated with oleylamine (OA–PB).

TGA of PB under nitrogen atmosphere up to ~800 °C showed three decomposition steps: I (30–224 °C), II (224–451 °C), and III (451–800 °C) (Figure 3). The mass loss in the first one (25.99%) was attributed to water loss from the PB structure. From these values, the ratio of water molecules per PB molecule was calculated to be 16. The mass decreases measured in steps II (14.12%) and III (18.06%) are mainly assigned to the cyanide groups released from the PB structure and the origination of iron carbides and graphite [33].

To check whether the synthesized PB was the insoluble form (Fe_4_[Fe(CN)_6_]_3_)·xH_2_O or also contained part of the soluble form (KFe[Fe(CN)_6_])·xH_2_O, the content of potassium and iron was determined using ICP. The wt % of K in PB was calculated to be 0.88, whereas the Fe was 28.39 wt %, indicating that PB is found mainly in the insoluble form.

### 2.2. Characteristics of OA–PB Nanoparticles

The insoluble PB was transformed into dispersible PB nanoparticles in organic solvents by means of the surface modification of PB using OA. OA formed a complex compound (OA–PB), with the Fe^III^–OH_2_ sites of the PB surface giving a deep-blue transparent solution in toluene. After evaporation of the organic solvent, the resulting residue gave unaltered XRD line widths (Figure S1), indicating that the size was unchanged in OA–PB nanoparticles. The UV/Vis absorption spectrum of a dilute dispersion of OA–PB gave an intense band at 677 nm, also owing to the charge transfer from Fe(II) to Fe(III). However, in comparison with the absorption of PB, a hypsochromic shift of ~23 nm was observed. This indicates that the excited state is more polar than the fundamental state, so it raises in energy in the less polar toluene solvent. To investigate the chemical content of OA–PB, TGA was conducted (Figure 3). Similarly to the TGA of PB, in the TGA of OA–PB, three stages are present. The first, between 30 and 296 °C, involved a mass loss of 27.48%, mainly owing to evaporation of water present in the PB structure. In the second step, between 286 and 479 °C, the mass loss raised up to 21.16%. Compared with the TGA of PB, the increase of mass loss in this step could be the result of the release of cyanide groups in gaseous form (cyanogen), as well as the degradation of OA. Pure OA is degraded at ~240 °C. The increase in the degradation temperature of OA–PB compared with pure OA is because the adsorbed OA is protected from degradation owing to their close proximity to the PB nanoparticles. The third weight loss, which represented a 13.09% loss, was observed between 479 and 799 °C. This reduction was a consequence of the complete decomposition of PB. Differences in the whole mass lost between PB (58.17%) and OA–PB (61.74%) pointed out that the content of OA in the nanoparticle was approximately 3.6% of the mass. The hydrodynamic diameter of OA–PB in toluene was 239.7 ± 6.3 nm, which is an average value from five measurements by dynamic light scattering (DLS). The polydispersity index was 0.359 ± 0.025. In this case, a monomodal distribution was obtained. The increase of hydrodynamic diameter of OA–PB in comparison with the value obtained with PB can be explained by the fact that the oleylamine layer coats a larger number of PB nanoparticles.

### 2.3. Characteristics of PB-Containing Lipid Nanocomplex (L–OA–PB)

A PB-containing lipid nanocomplex (L–OA–PB) was obtained by the thin film hydration method, as indicated in Section 3.4. After determining the size and polydispersity index of the preparations obtained at different times of shaking, the sample shaked for 48 h was the chosen, because it afforded the lowest polydispersity index and acceptable hydrodynamic radius (0.238 ± 0.00 and 93.8 ± 1.4, respectively; both determined by DLS (Table 1)).

**Figure 4:**
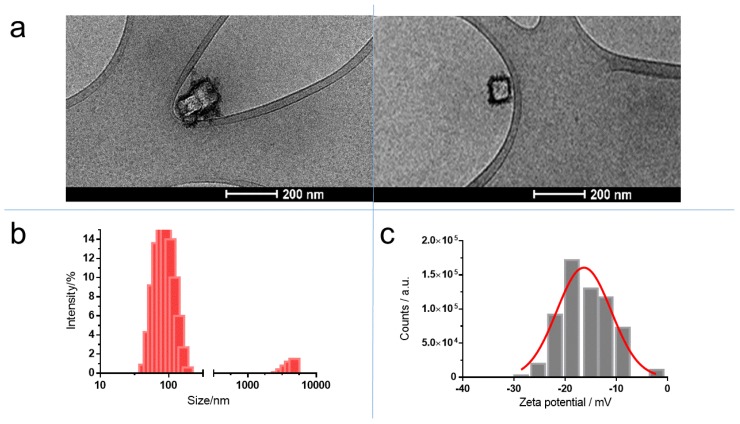
Characterization of PB nanoparticles trapped in lipid structure: (**a**) Cryo-TEM images; (**b**) size distribution determined by dynamic light scattering; and (**c**) ζ-potential distribution measured by Doppler electrophoresis.

The as-synthesized lipid nanocomplex was homogeneously dispersed in the aqueous solution and presented an intense blue color, indicating the presence of PB in the nanocomplex. Usually, the size distribution was monomodal, but in some preparations, a second peak appeared at ~4000 nm, representing <4% of the intensity (Figure 4b). By measuring the changes of size and polydispersity index, this nanocomplex was shown to be stable at three temperatures (4 °C, RT, and 37 °C) for a period of 45 days. No significant change in the above-mentioned parameters was observed. Cryo-TEM images did not show the classical circular structures observed when liposomes are present. As a special feature, some quadratic structures appeared (Figure 4a). This structure seemed to be coated by a layer of different material.

The lipid content was ≈ 20 mM phospholipid. The amount of PB was determined spectrophotometrically using the calibration curve obtained with PB nanoparticles. For this determination, a sample of 100 µL of lipid nanocomplex was diluted with 3 mL of Tris buffer pH 7.4, and the absorbance at 700 nm was determined. A value of 26 mg of PB (≈ 30.26 µmol) per mL of suspension was obtained. From the PB and lipid concentration, a PB loading of 1.3 g PB/mmol phospholipid (equivalent to 1.51 mol PB/mol phospholipid) was obtained.

**Figure 5:**
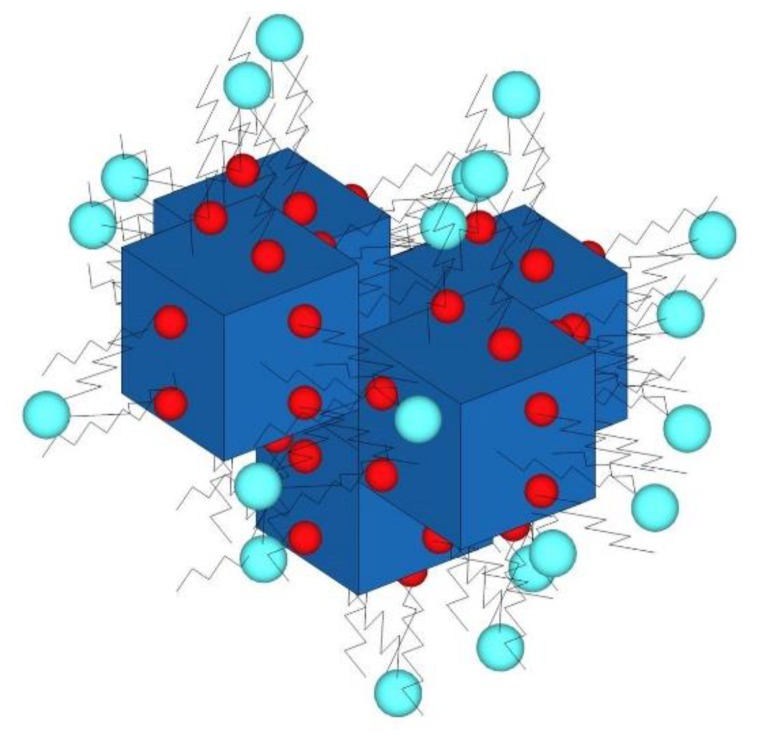
Schematic diagram of the as-synthesized lipid complex. Red: oleylamine (OA) polar head; blue: phospholipid polar head. The nucleus is formed by some individual particles of PB (blue). These are coated with molecules of OA (red), and the OA molecules are covered by phospholipids with the polar head at the external face (cyan).

The purification of the lipid complex with size-exclusion chromatography afforded only an eluate with a blue color, corresponding to the void volume where the nanoparticles were. This confirmed that PB nanoparticles were inside the lipid complex. The ζ-potential of these lipid structures after the shaking step of 48 h was –9.5 ± 0.3 (*n* = 5) (Figure 4c). The negative value of ζ-potential raises the possibility that OA molecules had undergone a displacement from the surface of PB aggregates to the medium, as OA bears a positive charge at neutral pH. It is known that aliphatic monoamines are able to form micellar structures at low critical micellar concentrations (cmc) [34]. For instance, stearylamine (with similar structure than OA, but without the double bond in the middle of the molecule) has a cmc of 67.6 µM [35]. Moreover, the ability of OA to act as a surfactant has been described previously [36]. To check the presence of OA in the lipid complex, a titration of the sample with fluorescamine was performed. Fluorescamine is a specific reagent for primary amines [37]. The results showed the presence of primary amines in the lipid product, because the fluorescence of this sample was significantly different from that obtained with a lipid sample prepared only with phospholipids. In consequence, the supposed displacement is not possible. As an explanation for the above data, we propose a complex formed by a nucleus of PB nanoparticles coated first by an inner monolayer of OA, as well as an outer layer of phospholipids (Figure 5).

## 3. Materials and Methods

### 3.1. Materials

Iron (III) chloride hexahydrate (FeCl_3_·6H_2_O), potassium hexacyanoferrite trihydrate (K_4_Fe(CN)_6_·3H_2_O), ammonium tyocyanate (NH_4_SCN), oleylamine (CH_3_(CH_2_)_7_CH=CH(CH_2_)_7_ CH_2_NH_2_), and fluorescamine were purchased from Sigma-Aldrich (St. Louis, MO, USA). Soybean phosphatidylcholine (S-100) was a gift from Lipoid (Ludwigshafen, Germany). Organic solvents (toluene, chloroform, butanol, methanol, and ethanol) were of analytical grade (Panreac, Barcelona, Spain). Acetone was of spectroscopic grade (MRW, Darmstad, Germany). Ultrapure water used in synthetic procedures was obtained from a Millipore Milli-Q system with a resistivity of ~18 MΩ cm.

### 3.2. Synthesis of Prussian Blue Nanoparticles

Insoluble nanoparticles of PB were obtained by precipitation as Fe_4_[Fe(CN)_6_]_3_·15 H_2_O. Briefly, 100 mL of an aqueous dispersion of FeCl_3_·6 H_2_O (6.48 g) and K_4_Fe(CN)_6_·3H_2_O (7.61 g) was mechanically stirred at RT for 15 min. The synthesis yielded a blue powder that was separated from the aqueous solution by centrifugation at 16,500 rpm for 60 min in a Digicen 21R centrifuge (Orto Alresa, Madrid, Spain). After the precipitation, the residue was rinsed three times alternatively with water and ethanol. Finally, the residue was dried under vacuum for 24 h at RT and the obtained powder was grinded with an agate mortar.

### 3.3. Synthesis of Hydrophobic Prussian Blue Nanoparticles

The hydrophobic OA–PB nanoparticles were prepared with slight variations according to the method described by Ishizaki et al. [38]. Briefly, the insoluble PB powder (1200 mg) was dispersed in 2.0 mL of water and the aqueous suspension was stirred for 24 h at RT. Then, 30 mL of a solution of n-butanol containing 240 mg of oleylamine was added to the previously prepared aqueous suspension and the mixture was stirred for three days at RT. After that, centrifugation was carried out (6000 rpm for 10 min in a Digicen 21R centrifuge) to separate the butanol and aqueous layers. The aqueous supernatant was discharged and the pellet was washed twice with diethyl ether to get rid of the non-reacting OA. To evaporate the diethyl ether, the residue was dried in air for 30 min, and, further, redispersed in toluene (6 mL). The toluene solution was stirred for three days at RT. Finally, the dispersion was centrifuged at 4000 rpm for 30 min. The resulting homogeneous blue dispersion containing OA–PB nanoparticles was kept at 4 °C for later use.

### 3.4. Formulation of Lipid Complex with Hydrophobic Prussian Blue Nanoparticles (L–OA–PB)

A lipid structure containing OA–PB (L–OA–PB) was obtained by the thin-film hydration method [39]. Briefly, soy-bean phosphatidylcholine (S-100) was dissolved in chloroform/methanol (2:1, *v*/*v*) at 20 mM concentration. Then, 4 mL of this solution was mixed with 4 mL of the toluene solution containing OA–PB. The resulting mixture was placed in a round-bottom flask and dried in a rotary evaporator (Büchi R-3000, Switzerland) under reduced pressure at 35 °C, until a thin blue film was formed at the inner surface of the flask. Afterwards, the resulting lipid–OA–PB film was hydrated with 4.0 mL of Tris buffer (10 mM, pH 7.4). The dispersion was sonicated in bath sonicator (Transsonic Digital Bath sonifier, Elma, Singen, Germany) for 5 min. After that, the lipid suspension was constantly shaken several times (from 1 h to 60 h) at 30 rpm in a test tube rotator (L-29, Labinco, Breda, The Netherlands) at RT. The resulting lipid structures presented an intense blue color.

### 3.5. Characterization of PB, OA–PB, and L–OA–PB

Transmission electron microscopy (TEM) (Jeol 1010 microscope, Tokyo, Japan) was used to characterize PB, OA–PB, and L–OA–PB operating at 80 kV. A drop of the sample was placed over the surface of a copper grid, previously covered with a thin amorphous carbon film, for 1 min. Then, the excess solution was removed with an inert paper. In the L–PB, the sample was stained previously with uranyl acetate. The grids were then dried in air. A Megaview III camera was used to record the images, with the acquisition then being accomplished with a Soft-Imaging Software (SIS, Hamburg, Germany). The morphology of L–OA–PB was analyzed by Cryo-TEM. For these observations, grids were transferred to a Tecnai F20 (FEI, Eindhoven, The Netherlands) by means of a cryoholder (Gatan, Warrendale, PA, USA). Images were taken at 200 kV in a temperature range from −175 to −170 °C using low-dose imaging conditions with a 4096 × 4096 pixel CCD Eagle camera (FEI, Eindhoven, The Netherlands). Dynamic light scattering (Zetasizer S90, Malvern, UK) was used to determine the size and size distribution of the different nanoparticles. The ζ-potentials were determined by Doppler microelectrophoresis (Zetasizer Nano SZ, Malvern, UK) after dispersing the sample in KBr 10^−3^ M. X-ray diffraction (XRD) patterns were used in PB to identify the crystallite phase. The equipment used was a Bragg-Brentano θ/2θ Siemens D-500 diffractometer (radius = 215.5 mm) equipped with a Cu Kα radiation source. The amount of water and OA coating in PB and OA–PB, respectively, were quantified by thermogravimetric analysis (TGA) using a TGA/SDTA851e (Mettler Toledo, Toledo, OH, USA) with a 10 °C/min heating rate under a nitrogen atmosphere (50 mL · min^−1^). The measurement was recorded from RT up to 800 °C. The UV/Vis absorbance spectra of as-synthesized nanoparticles were obtained between 350 and 850 nm using a Shimadzu UV-2401PC spectrophotometer (Shimadzu, Tokyo, Japan). PB colloidal solutions were obtained by mixing 1 mg of PB in 10 mL of water. The content in iron and potassium was determined with an inductively coupled plasma optical emission spectroscope (ICP-OES, Perkin Elmer Optima 3200RL, Reston, VA, USA). The lipid amount in the complexes was determined by the Steward–Marshall method [40]. OA was measured spectrofluorometrically using fluorescamine as a labeling reagent [34]. The stability of L–OA–PB was determined by measuring the changes in size with time of the samples kept at RT and at 4 °C.

## 4. Conclusions

In summary, we designed and synthesized a new kind of about 100 nm hybrid nanoparticles formed by PB in colloidal form stabilized with phospholipids through a thin-film hydration process. The coating of PB with phospholipids produced a nanosystem fully biocompatible and dispersible in aqueous media. The presence of phospholipids enhanced the biocompatibility and colloidal stability of the nanocarrier. The hydrocarbon chains of the lipid provide the chance to encapsulate hydrophobic drugs. Moreover, the phospholipid surface could be modified with diverse functional groups, such as peptides or fluorescent probes. According to the properties of PB, these particles can be used as a photothermal platform or as an adsorbent agent for cations.

## Figures and Tables

**Table 1:** Evolution of size and polydispersity index of lipid (L)–oleylamine (OA)–Prussian blue (PB) over time (*n* = 5).

Time/h	Size/nm	Polydispersity Index
1	200 ± 8.19	>1
2	210 ± 6.11	0.889 ± 0.015
12	160 ± 4.18	0.750 ± 0.028
24	105 ± 5.20	0.700 ± 0.009
48	93.8 ± 1.40	0.238 ± 0.009
60	94.0 ± 1.25	0.225 ± 0.010

## Supplementary Materials

The following are available online, Figure S1: XRD determination of PB nanoparticles coated with oleylamine.
