# Supplementary material for: Facile Synthesis of Novel Prussian Blue–Lipid Nanocomplexes
Source: Molecules. 2019 Nov 15;24(22):4137. doi: 10.3390/molecules24224137 (PMC6891449; doi:10.3390/molecules24224137)
Supplement: Supplementary file 1 [file molecules-24-04137-s001.pdf]

Supporting information

Facile synthesis of novel Prussian blue-lipid nanocomplexes

M. Antònia Busquets[1,2], Ariadna Novella-Xicoy[1], Valeria Guzmán[3], and Joan Estelrich[1,2,*]

[1]Pharmacy and Pharmaceutical Technology and, Physical Chemistry Department. Faculty of Pharmacy and Food Sciences. University of Barcelona. Avda. Joan XXIII, 27-31. 08028 Barcelona. Catalonia. Spain.
[2]Institute of Nanoscience and Nanotechnology. IN[2]UB. Diagonal 645. 08028 Barcelona. Catalonia. Spain.
[3]Polytechnic University of Sinaloa. Carretera Municipal Libre Mazatlán Higueras Km 3, 82199 Mazatlán, Sinaloa, Mexico.
*Correspondence: joanestelrich@ub.edu; Tel.: +34.934 024 559

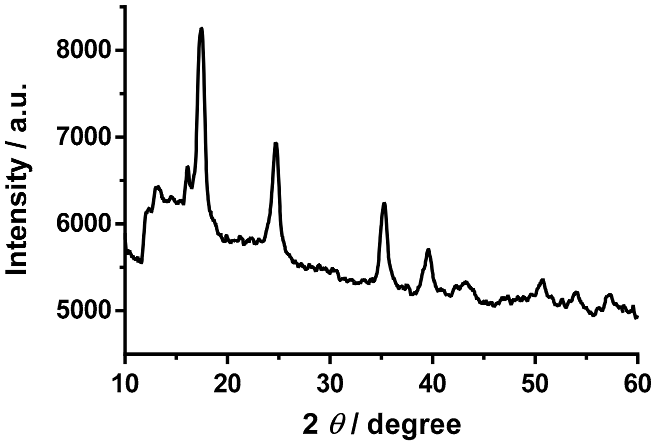

Figure S1. XRD determination of PB nanoparticles coated with oleylamine.
